# γ Radiation Effects on Transition Metal Dichalcogenides: A Review of Defect Mechanisms and Device Implications

**DOI:** 10.1021/acsomega.5c13158

**Published:** 2026-03-14

**Authors:** Diogenes Kreusch, Fernando M. Araujo-Moreira

**Affiliations:** 28098 Instituto Militar de Engenharia, Rio de Janeiro 22290-270, Brazil

## Abstract

Two-dimensional (2D) transition metal dichalcogenides (TMDs) are foundational materials for next-generation electronics. For their viable use in space, nuclear, and medical applications, a comprehensive understanding of their response to high-energy ionizing radiation is critical. This review synthesizes and critically analyzes 32 experimental studies published since 2016 to build a unified framework for γ-radiation effects in TMDs. We found that the primary defect mechanism is the creation of chalcogen vacancies (CVs). The subsequent material response is decisively modulated by the irradiation environment: in ambient air, CVs are passivated, leading to oxidation, whereas in a vacuum or inert gas, pure vacancy effects dominate. This dichotomy resolves apparent contradictions in the literature regarding electronic doping; for instance, WSe_2_ consistently exhibits intrinsic n-type doping (from V_Se_), while WS_2_ and MoS_2_ in air trend toward p-type doping (from charge-transfer to oxides). These competing mechanisms of doping, strain, and disorder are mapped to their complex spectroscopic signatures in Raman and photoluminescence, including nonmonotonic dose-dependence. Beyond damage, γ-rays are shown to be a potent tool for defect engineering, inducing emergent properties such as room-temperature ferromagnetism (attributed to V_1M+2S_ complex vacancies) and optimizing catalytic activity. Finally, this review finds that TMD-based devices possess high intrinsic radiation hardness, with most failures originating from charge trapping in the adjacent dielectrics, not the 2D channel. We conclude by identifying critical gaps in the literature, including the unexplored effects of dose rates and the need for *in situ* characterization.

## Graphical abstract

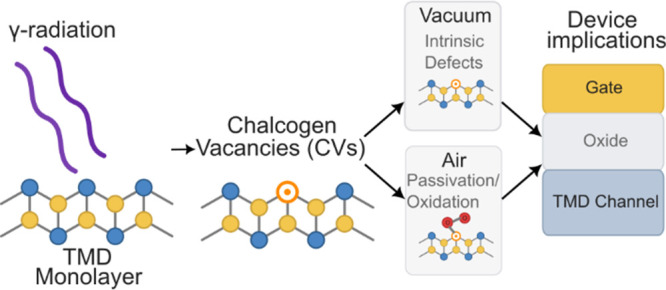

Since the isolation of graphene, two-dimensional materials (2DMs) have emerged as a versatile platform for applications in electronics,^1^ optoelectronics,^2^ sensing,^3^ and catalysis,^4^ owing to their distinctive electronic and optical properties such as high charge-carrier mobility and strong light–matter interactions.^5^

Among these 2DMs, transition metal dichalcogenides (TMDs) figure as solids with the chemical formula MX_2_, where “M” stands for a group VI metal, such as tungsten (W), molybdenum (Mo), etc., and “X” stands for a chalcogen, such as sulfur (S), selenium (Se), tellurium (Te), etc., and are among layered van der Waals solids. Notably, MoS_2_, WS_2_, and WSe_2_ stand out among 2DMs due to their monolayer direct band gaps, high transistor ON/OFF ratios, and tunable light absorption across visible and near-infrared wavelengths.^6^ Their versatile properties have enabled applications as room-temperature single-photon emitters, high-performance field-effect transistors, photodetectors, flexible electronics, chemical and biological sensors, and catalysts, as well as emerging roles in valleytronics and spintronics, positioning TMDs as a cornerstone for next-generation technologies that require robust operation under harsh conditions.^7^ To bridge the gap between laboratory demonstrations and practical deployment, it is, therefore, imperative to assess how TMD-based devices withstand ionizing radiation in demanding environments.

While semiconducting TMDs such as MoS_2_, WS_2_, MoSe_2_, and WSe_2_ have received the most attention due to their direct band gaps and relevance for logic and optoelectronic devices, the TMD family also includes metallic compounds, such as PtSe_2_, which exhibit distinct electronic structures and transport mechanisms. These materials are increasingly explored for applications in sensing, contacts, and radiation-tolerant devices, where carrier transport is less sensitive to bandgap modulation but can be strongly affected by defect formation, charge trapping, and dielectric interactions.^8^ Consequently, understanding γ-radiation effects across the full spectrum of TMD electronic phases is essential for assessing both fundamental radiation–matter interactions and device-level robustness.

In particular, γ-rays (E > 100 keV) pose a significant challenge by interacting with materials via photoelectric absorption, Compton scattering, and pair production. These processes generate energetic electrons that displace lattice atoms, creating vacancies, interstitials, and defect cascades. Other types of ionizing radiation (β and α particles, protons, etc.) can be easily shielded when compared to γ-rays. Such structural damage can degrade the electrical, optical, and mechanical integrity of TMDs, calling for a detailed understanding of γ-induced effects to guide the design of radiation-tolerant two-dimensional electronics.^9^

Although research on radiation effects in 2DMs has advanced, dedicated studies on γ irradiation remain scarce. Nevertheless, Walker et al.^9^ provided a seminal review compiling contributions on γ-ray, X-ray, low-energy electron, proton, and heavy-ion irradiation of 2DMs, shedding light on damage mechanisms and material responses. In their review, prior work on γ-ray interactions in two-dimensional materials is compiled, noting that Compton-scattered electrons in graphene can displace carbon atoms to generate vacancies and interstitials, a process that Raman spectroscopy studies have shown saturates structural defects at high doses. The review further describes how X-ray and γ-ray exposure produces Dirac-voltage shifts in graphene field-effect transistors, a sensing effect attributed to radiation-induced charges altering the local electric field. As for TMDs, the review compiles theoretical density functional theory (DFT) results indicating that exposure to γ-rays could introduce deep-level traps and induce a direct-to-indirect bandgap transition in WSe_2_, as well as generate sulfur vacancies (S-vacancies) in MoS_2_ that behave as deep acceptors or shallow donors, thus modifying their electronic characteristics. It is important to note, however, that these conclusions lacked specific experimental validation under γ irradiation at the time. While other reviews have been published, such as the work by Zhao et al.,^10^ their scope was restricted to two-dimensional 2H – MoS_2_ few layers, excluding other TMDs and diverse nanostructured forms from consideration.

Notably, until 2016, dedicated experimental studies on γ-ray effects in transition-metal dichalcogenides were very limited; therefore, this review surveys post-2016 research on γ-ray interactions with TMDs to guide the development of radiation-hardened two-dimensional electronics and quantum devices.

## Results

Following the systematic search methodology detailed in the Methods section, a total of 32 peer-reviewed studies published since 2016 were selected for this review. These articles form the basis for analyzing the current understanding of γ-radiation effects on transition metal dichalcogenides.

A comprehensive summary and direct comparison of these 32 studies are presented in Table 1. This table compiles the critical experimental parameters from each study, including the specific TMD material (MoS_2_, WSe_2_, etc.), material form factor (e.g., monolayer, thin film, nanosheet), irradiation environment (ambient air, vacuum, Ar), irradiation dose rate, total ionizing dose (TID), γ-ray source, and key findings for each study. Units were standardized to enable study comparisons.

**Table 1:** Summary of Experimental Parameters and Properties Investigated in the Selected Studies on γ-irradiated Transition Metal Dichalcogenides

**ref.**	**TMD**	**material form**	**environment**	**dose rate/source activity**	**TID**	**source isotope**	**key findings**
^11^	MoS_2_	few-layers on Si/SiO_2_ substrate FET	ambient air	2.185 and 18.83 kGy/h	6.2, 23.5, 31.2, 37.5, and 100 kGy (Si)	Co-60	High radiation tolerance [up to 100 kGy (Si)] in PSE-gated FETs; tolerance is repairable by thermal anneal and can be enhanced by preirradiation.
^12^	MoS_2_	thin film	ambient air	2.09 kGy/h	1500 kGy	Co-60	High dose caused S-vacancies, oxidation, grain coalescence, and an indirect band gap shrinkage from 1.50 to 1.41 eV.
^13^	MoS_2_	nanosheets	ambient air	Not reported	10, 100, 300, 500, and 800 kGy	Co-60	Strain from S-vacancies rises with dose up to 300 kGy then decreases; Raman peaks show a corresponding blue shift followed by a red shift.
^14^	MoS_2_	multilayer nanomechanical resonator	vacuum	Activity of 3.7 × 10^–2^ GBq	Exposure over 12 or 24h	Cs-137	Ultrasensitive γ responsivity (0.02–0.05 photon detection limit) in nanomechanical resonators due to charge accumulation and tensioning.
^15^	MoS_2_	monolayer on copper substrate	not reported	0.124 kGy/h	1.0, 1.75, 2.65, and 3.0 kGy	Co-60	Raman shifts indicate S-vacancy-induced compressive strain and point defects; confirm vacancy engineering on copper substrates without transfer artifacts.
^16^	MoS_2_	monolayer on PVA/glass, PMMA/glass and Si/SiO_2_ substrate	ambient air	2.316 kGy/h	1, 5, 10, 20, and 40 kGy	Co-60	MoS_2_ monolayers are radiation resistant up to 40 kGy; photoluminescence shows a dose-dependent trion (X band) signal that is most intense at 5 kGy and disappears at 40 kGy.
^17^	MoS_2_	few-layer and bulk films on Si/SiO_2_ substrate	ambient air	0.9 kGy/h	3, and 9 kGy	Co-60	Induced ultrahigh RTFM (≈610 *emu*/*cm* ^3^) in few-layer (3.5 nm) MoS_2_ films; bulk (40 nm) films were unaffected. Magnetism is attributed to V_1Mo+2S_ complex vacancies.
^18^	MoS_2_	few layers on Si/SiO_2_ substrate	ambient air	Not reported	1200 kGy	Co-60	An ultrahigh dose caused the complete conversion of MoS_2_ to MoO_*x*_, evidenced by the disappearance of characteristic E_2g_^1^ and A_1g_ Raman modes.
^19^	MoS_2_	monolayer on nickel-coated copper substrate	not reported	0.115 kGy/h	1.92, 2.65, 2.92, 3.0, 3.67, 5.3, and 6.0 kGy	Co-60	Defects evolve from S-vacancies at low doses to include Mo-related vacancies at higher doses; DFT calculations show progressive bandgap narrowing with increased vacancy concentration.
^20^	MoS_2_	nanosheets	ambient air	3.6 kGy/h	1, 10, 100, and 1000 kGy	Co-60	S-vacancies enhance HER catalytic activity, with an optimal dose at 100 kGy; higher doses (1000 kGy) degrade performance due to excessive disorder and oxidation.
^21^	MoS_2_	mono- and few-layer and bulk layered on the Si/SiO_2_ substrate	ambient air with 95% RH	15.9–21.9 kGy/h	100, 500, and 1000 kGy	Co-60	Induces p-doping in monolayer MoS_2_ (trion dissociation, A_1_^′^ blue shift). High doses (>500 kGy) cause edge-selective radiolytic etching and oxidation, enhanced by surface water.
^22^	MoS_2_	thin film on Al_2_O_3_ substrate FET	ambient air	1 kGy (Si)/h	1, 2, and 3 kGy (Si)	Co-60	Caused positive V_th_ shifts and a 15.7% I_on_ decrease in FETs. Degradation is attributed to hole trapping in the Al_2_O_3_ dielectric, not the MoS_2_ channel.
^23^	MoS_2_	thin film on Si/SiO_2_ substrate phototransistor	ambient air (CYTOP-coated)	not specified	0.2, 0.4, 0.8, and 20 kGy	Co-60	Phototransistor degradation (mobility, responsivity drop) is dominated by positive charge build-up in the SiO_2_ gate dielectric. QD encapsulation enhances radiation hardness.
^24^	MoS_2_	nanoflakes	water dispersion	not specified	4.84 × 10^–4^ kGy (in the entire vial)	Ga-68	At sub-Gy doses in a water solution, MoS_2_ oxidation is driven indirectly by water-radiolysis products (e.g., OH• radicals), not direct irradiation.
^25^	MoS_2_	monolayer on sapphire substrate	ambient air	3.2 kGy/h	1, 5, 10, 55, 130, 275, 500, 725, and 1000 kGy	Co-60	Monolayer MoS_2_ is radiation hard up to ≈130 kGy. Higher doses induce S-vacancy formation, progressive oxidation, and edge etching.
^26^	MoS_2_	monolayer on Al_2_O_3_ substrate FET	ambient air	1.60 kGy/h	10 kGy	Co-60	MoS_2_ memtransistors and logic gates show high radiation resilience up to 10 kGy; functionality is preserved despite modest V_th_ drift and mobility decrease.
^27^	MoS_2_	nanosheets	ambient air	3.76 kGy/h	1, 10, 100, and 1000 kGy	Co-60	Showed a complex, nonmonotonic conductivity dependence on both dose and temperature. At *T* < 100 °C, conductivity peaked at 10 kGy (a 6.2x increase at 25 °C); at *T* > 100 °C, the trend reversed, and conductivity hit a minimum at 1 kGy. Irradiation improved crystallinity and decreased grain size, causing a red shift in both E_2g_^1^ and A_1g_ Raman modes. XPS confirmed no oxidation or change in stoichiometry.
^28^	MoSe_2_	nanoflakes on Si/SiO_2_ substrate	ambient air	1.62 kGy/h	10, 50, 80, and 100 kGy	Co-60	MoSe_2_ flakes show high resilience up to 100 kGy with only minor optical degradation; PL/XPS shifts suggest vacancy healing via Mo adatom migration.
^29^	MoSe_2_ and WS_2_	monolayer on Si/SiO_2_ substrate	vacuum and Ambient air	Activity of 1.04 GBq and 0.96 GBq	Exposure over 2h27	Na-22	WS_2_ and MoSe_2_ devices are resilient to space-relevant doses. In air, WS_2_ PL increases due to oxygen passivation of S-vacancies; this effect vanishes in vacuum.
^30^	WS_2_	nanopowder	ambient air (embedded in bioactive glass)	MCNP simulation	-	-	WS_2_ nanopowder in bioactive glass composites increases mass attenuation coefficients, acting as an effective γ-ray shielding material.
^31^	WS_2_	nanosheets	ambient air	1.75 kGy/h	12, 34, 48, and 96 kGy	Co-60	Induces exciton-to-trion conversion, a 3-fold rise in spin density (from S-vacancies), and progressive, dose-dependent exfoliation (thinning) of WS_2_ nanosheets.
^32^	WS_2_	monolayer on sapphire substrate	ambient air	6.6 kGy/h	1, 50, 100, 200, and 400 kGy	Co-60	Induces p-type doping in monolayer WS_2_, causing trion-to-exciton conversion and a 10-fold PL intensity increase. Doses >50 kGy cause etching due to thermal strain.
^33^	WS_2_	monolayer on Si/SiO_2_ substrate	sealed in acrylic box (environment inside not reported)	2.7 kGy/h	0.1, 0.2, 0.4, 0.8, and 1.0 kGy	Co-60	Induces effective p-type doping (trion enhancement, A_1g_ blue shift) and a magnetic phase transition (RTFM) at 0.4 kGy, attributed to V_1W+2S_ complex vacancies.
^34^	WS_2_	few layers on the FTO substrate	ambient air	1.2 kGy/h	1, 25, 50, 75, and 100 kGy	Co-60	S-vacancy creation and ambient oxidation in WS_2_ films narrow the band gap (1.64 to 1.57 eV) and increases saturation current by 1000-fold at 100 kGy.
^35^	WS_2_ and WSe_2_	monolayer on Si/SiO_2_ substrate	vacuum	0.845 kGy(Si)/h	142, 284, and 426 kGy (Si)	Co-60	In high vacuum, WSe_2_ and WS_2_ show high radiation hardness. WSe_2_ PL progressively increased with dose, while WS_2_ PL enhancement was transient.
^36^	WSe_2_	mono- and few-layers nanoflakes on sapphire substrate	ambient air	activity of 22.57 GBq	30 kGy	Co-60	Trilayer WSe_2_ shows the lowest friction. γ-rays passivate Se-vacancies with oxygen (reducing trap states) and ″clean″ organic adsorbates from the surface.
^37^	WSe_2_	monolayer on sapphire substrate	ambient air	activity of 22.57 GBq	5, 10, 15, and 20 kGy	Co-60	Induces n-type doping (via oxidation) and a tightly bound negative trion (X^–^) with a large ≈90 meV binding energy.
^38^	WSe_2_	monolayer on sapphire substrate	argon	activity of 22.57 GBq	20, 40, 60, 80, and 100 kGy	Co-60	Induces n-type doping (from Se-vacancies) and a direct-to-indirect bandgap transition. Raman shifts are linearly dependent on vacancy content.
^39^	WSe_2_	thin films on FTO substrate	ambient air	not mentioned	1, 10, and 100 kGy	Co-60	S-vacancies and oxygen passivation in WSe_2_ films reduce the band gap (1.60 to 1.14 eV) and cause a near-linear increase in current with dose.
^40^	WSe_2_	nanosheets	vacuum	1.75 kGy/h	12, 34, 48, and 96 kGy	Co-60	Creates chalcogen-vacancy defects in WSe_2_, leading to stronger trion signatures, a new defect band near 790 nm, and longer PL decay times (trap-mediated pathways).
^8^	PtSe_2_	nanograins on nanolayer on sapphire substrate	N_2_ with varying RH	0.5 kGy/h	10 kGy	Co-60	PtSe_2_ humidity sensor shows high radiation tolerance, exhibiting identical electrical and sensing performance after a 10 kGy dose.
^41^	MoTe_2_	thin film	ambient air	6.6 kGy/h	10, 50, 100, 200, 400, and 600 kGy	Co-60	Induces p-type doping (via V_Te_ creation and subsequent oxidation in air); work function increased by 0.14 eV. Raman modes (A_1g_ and E_2g_^1^) show red shifts due to tensile strain. Films stable up to 50 kGy; etching observed ≥ 100 kGy.

The following sections provide a thematic synthesis of the collective findings from these studies, beginning with an analysis of the most commonly identified defect mechanism.

### The Primary Defect Mechanism: Chalcogen Vacancies and Oxidation

A strong consensus across the post-2016 literature identifies the primary defect-creation mechanism in TMDs as the formation of chalcogen vacancies (CVs).^12,13,15,19–21,25,31,32,34,36–38^ High-energy γ photons interact with the material, primarily via Compton scattering, to generate energetic secondary electrons. These electrons, in turn, can displace the lighter chalcogen atoms from the lattice via knock-on damage when their transferred energy exceeds the displacement threshold;^9^ the probability of which rises with dose and is strongly modulated by pre-existing defects and edges.

The subsequent chemical evolution of these vacancies is critically dependent on the irradiation environment, a finding that cleanly separates the primary displacement damage from the secondary chemical reactions. When irradiation is conducted in ambient air, the highly reactive vacancy sites are passivated by oxygen and water. This is consistently reported as leading to the formation of metal–oxygen bonds (Mo–O or W–O),^18,32,39^ metal oxides (MoO_*x*_ or WO_3_),^12,18,25,37,39^ and, in some cases, sulfate (SO_4_^2–^) species.^21,31,34^ At extreme doses, this process can be destructive; Ozden et al.^18^ observed the complete conversion of MoS_2_ to MoO_*x*_ after a 1200 kGy dose, a TID far exceeding those used in most device-centric studies, and Isherwood et al.^21^ detailed a full reaction sequence from oxysulfides to Mo^6+^ oxides.

In contrast, studies performed in high vacuum^29,35^ or an inert argon atmosphere^38^ isolate the effects of the vacancies themselves, provided subsequent exposure to ambient air is limited. These studies report that in the absence of oxygen and water, the TMDs exhibit minimal chemical alteration. Vogl et al.^29^ provided a direct comparison, noting that the effects observed in WS_2_ irradiated in air vanished when the experiment was repeated in a vacuum. Similarly, Wu et al.^38^ confirmed that in an Ar environment, selenium vacancies are generated without the subsequent oxidation, demonstrating that the ambient atmosphere is a key participant in the defect mechanism.

While simple CVs are the most commonly cited defect, several studies propose the formation of more complex defects. Anbalagan et al.^17^ and Felix et al.^33^ both attribute the emergence of ferromagnetism to complex vacancies involving both a metal atom and adjacent chalcogens (V_1Mo+2S_ and V_1W+2S_, respectively). Chavda et al.^19^ also suggest an evolution from purely S-vacancies at low doses to the inclusion of Mo-related vacancies at higher doses. Finally, a separate indirect mechanism was identified by Singh et al.^24^ for MoS_2_ irradiated in a water solution, where oxidation was driven not by direct radiation but by radiolysis products, such as OH• radicals.

### Dose-Dependent Effects: From Enhancement to Degradation

The total ionizing dose is a critical parameter in all 32 reviewed studies, and the collected data reveal that its effects are complex and often nonlinear. The outcomes can be broadly categorized into dose-dependent thresholds, linear scaling, nonmonotonic “turn-around” effects, and high-dose degradation.

Several studies establish a linear or monotonic dependence between the dose and a given effect. For instance, the Raman shifts and broadening in γ-irradiated WSe_2_ were found to be linearly dependent on the vacancy and oxidation content, which in turn scales with the applied dose.^38,39^ Similarly, electrical conductivity in WS_2_ ^34^ and WSe_2_ ^39^ films was observed to increase progressively with dose, as more defects were introduced. This progressive, dose-dependent change was also seen structurally, with Sarmah et al.^31^ reporting that γ-irradiation acts as a “thinning″ tool, progressively reducing the average layer count of WS_2_ nanosheets as the dose increased.

Other studies identify clear thresholds for damage. Singh and Singh^25^ delineated a “hardness window” for monolayer MoS_2_, finding it remains largely intact up to ≈130 kGy, after which progressive oxidation and etching begin. A similar threshold was noted by Aggarwal et al.,^32^ who observed significant etching only at doses of 50 kGy and above. Saturation effects were also reported; Chen et al.^11^ found that threshold-voltage shifts in MoS_2_ field-effect transistors (FETs) were pronounced during the initial 6.2 kGy­(Si) exposure but evolved much more slowly at subsequent doses.

More complex, nonmonotonic “turn-around” effects were also observed, suggesting a competition between defect-creation and other mechanisms. A clear example is seen in catalysis, where Dong et al.^20^ found an optimal dose of 100 kGy for enhancing the hydrogen evolution reaction (HER) in MoS_2_; excessive doses beyond this point degraded performance due to extensive disorder and oxidation. This “turnaround” behavior was also reported spectroscopically. Kolhe et al.^13^ noted that out-of-plane strain in MoS_2_ films increased up to 300 kGy but then diminished at higher doses (500–800 kGy), with Raman peaks showing a corresponding blue shift followed by a red shift. Garcia^16^ observed a similar nonmonotonic trend in MoS_2_ photoluminescence, where the trion signal was maximized at a low dose (5 kGy) but disappeared at a higher dose (40 kGy).

At extremely high doses, the effects are consistently destructive, leading to significant structural and chemical transformation. Doses of 400 kGy,^32^ 1000 kGy,^21^ 1200 kGy,^18^ and 1500 kGy^12^ were all reported to cause severe etching, progressive bond degradation, sulfur sublimation, and, in the most extreme case, the complete removal of the monolayer or its full conversion to MoO_*x*_.

### Spectroscopic Signatures: Modulating Photoluminescence and Raman Modes

The primary tools used to characterize the electronic and structural changes in irradiated TMDs are photoluminescence (PL) and Raman spectroscopy. These techniques provide a nondestructive “fingerprint″ of the material, revealing the direct consequences of defect-induced doping, strain, and disorder.

The primary optical consequence of γ-irradiation is the modulation of carrier concentration (doping), which is directly observed in the PL spectra as a change in the balance between neutral excitons (X^0^, an electron–hole pair) and charged excitons (trions, X^–^ or X^+^).^31–34,37^

The findings are highly material-dependent. In WSe_2_, irradiation consistently induces n-type doping (an excess of electrons), which leads to the emergence and strengthening of the negative trion (X^–^) peak. Wu et al.^37^ specifically identified this as a “tightly bound” trion with a large binding energy of ≈90 meV.

Conversely, in WS_2_ and MoS_2_, which often become p-doped upon irradiation in air, a trion-to-exciton conversion (X^–^ to X^0^) is observed. This suppression of the trion population, combined with the passivation of vacancy-related nonradiative recombination sites by oxygen, can lead to a significant increase in the overall PL intensity.^21,32,34^

However, the effect is complex and not always linear. Other studies on WS_2_ ^31,33^ reported an enhancement of the trion emission, not its suppression, which was also attributed to p-doping. Furthermore, Garcia^16^ found a nonlinear effect in MoS_2_, where a low 5 kGy dose maximized the trion signal, but a higher 40 kGy dose caused it to disappear entirely. At sufficiently high doses (e.g., 1500 kGy), any nuanced excitonic effects are overshadowed by overwhelming defect creation, leading to a marked drop in PL intensity and reduced crystallinity.^12^

Raman spectroscopy, which probes the material’s vibrational modes, is highly sensitive to the strain, doping, and disorder introduced by irradiation. The two primary modes analyzed are the in-plane E_2g_^1^ and out-of-plane A_1g_ modes.

The findings for WSe_2_ are remarkably consistent: irradiation causes a blue shift (stiffening) of the A_1g_ mode and a red shift (softening) of the E_2g_^1^ mode. Both shifts were found to be linearly dependent on the vacancy/oxidation content, providing a potential method for quantifying defect density.^37^

The results for WS_2_ and MoS_2_ are more complex and contradictory, reflecting a competition between different physical mechanisms: for WS_2_, some studies report a blue shift of the A_1g_ mode, which is consistent with the p-doping effect (electron–phonon coupling).^33^ In contrast, others report a red shift in both the E_2g_^1^ and A_1g_ modes, attributing it to strain and disorder caused by S-vacancies.^32,34^ As for MoS_2_, the results are even more varied. Kolhe et al.^13^ observed a blue shift at low doses (up to 300 kGy) that transitioned to a red shift at high doses (500–800 kGy). Other studies have reported p-doping-induced blue shifts, nonmonotonic shifts, or shifts driven by strain. Singh et al.^24^ found that irradiation in water caused red shifts, linking the effect to the weakening of interlayer forces.

At extreme doses, the lattice is irreversibly damaged. Ozden et al.^18^ demonstrated that after a 1200 kGy dose, the characteristic E_2g_^1^ and A_1g_ Raman peaks of MoS_2_ almost disappeared, confirming the material’s complete conversion to molybdenum oxide.

### Emergent Properties

Beyond modulating existing electronic and optical properties, γ-irradiation can be employed as a tool to induce entirely new functionalities in TMDs, including magnetism, enhanced catalytic activity, and unique mechanical responses.

Perhaps, the most striking emergent property is the induction of room-temperature ferromagnetism (RTFM) in inherently nonmagnetic (diamagnetic) TMDs. Two key studies have demonstrated this magnetic phase transition: Anbalagan et al.^17^ observed ultrahigh RTFM (M_s_ ≈ 610 emu/cm^3^) in few-layered MoS_2_ films after a 9 kGy dose, and Felix et al.^33^ reported a similar diamagnetic-to-ferromagnetic transition in monolayer WS_2_ at a specific dose of 0.4 kGy, resulting in a hysteresis loop with coercivity of 37 Oe.

In both cases, this emergent magnetism was attributed to complex vacancies rather than simple chalcogen vacancies, specifically, V_1Mo+2S_ in MoS_2_ and V_1W+2S_ in WS_2_. This effect was also found to be strongly thickness-dependent; Anbalagan et al.^17^ noted that while few-layer MoS_2_ became strongly ferromagnetic, bulk MoS_2_ remained completely diamagnetic after the same irradiation dose.

Relatedly, irradiation was shown to modify the spin dynamics. Sarmah et al.^31^ used electron paramagnetic resonance to reveal a 3-fold rise in spin density in WS_2_ nanosheets, which they ascribed to S-vacancies promoting a uniform spin distribution, highlighting a potential pathway for spintronic applications.

Gamma irradiation has also been used as a tool for mechanical and structural engineering. The effects on friction, however, appear to be highly dependent on the irradiation environment. Wu et al.^38^ found that in an argon environment, irradiation-induced vacancies led to a monotonic increase in friction with dose. In contrast, a separate study by Wu et al.^36^ in ambient air found that irradiation could reduce friction, attributing this to a “cleaning″ of the material surface and the passivation of vacancy trap states by oxygen.

At higher doses, irradiation acts as a “thinning″ or “etching″ tool. Sarmah et al.^31^ reported progressive exfoliation in WS_2_ nanosheets, where the average layer count was reduced from 26 to 10 at the highest dose. At higher doses (≥50 kGy), this effect becomes destructive, leading to etching of layers and, ultimately, the complete removal of films at doses ≥ 400 kGy.^32^

The controlled creation of defects has been leveraged for specific applications. Dong et al.^20^ demonstrated that γ irradiation can precisely tune the hydrogen evolution reaction (HER) catalytic activity of MoS_2_ nanosheets. They identified an optimal dose of 100 kGy that created the ideal S-vacancy density to enhance catalytic sites and lower the overpotential, while excessive doses degraded the performance.

Beyond that, γ-irradiation has enabled novel device functions. For example, as demonstrated by Deliormanlı et al.,^30^ WS_2_ nanopowder can be useful for radiation shielding when embedded as a composite in bioactive glass, elevating mass attenuation coefficients and serving as an effective shield against ionizing radiation. Furthermore, Lee et al.^14^ have shown that TMDs subjected to irradiation exhibit ultrasensitive capabilities: MoS_2_ nanomechanical resonators are so sensitive to ionization-induced charge and tensioning that they can be used for low-dose photon sensing, with a detection limit as low as 0.02–0.05 photons.

### Device Performance, Hardness, and Radiation Tolerance

The inherent robustness of TMDs against radiation damage, combined with their novel electronic properties, makes them prime candidates for radiation-hardened electronics. The reviewed studies collectively demonstrate that while TMD-based devices are highly tolerant, their performance is often limited by the surrounding dielectric materials rather than the 2D channel itself.

TMD-based devices exhibit remarkable radiation tolerance across a range of doses. Schranghamer et al.^26^ demonstrated that MoS_2_ memtransistors and logic gates (NOT, AND, NAND, etc.) remained fully functional with only modest material and device changes after a 10 kGy TID. Similarly, Chen et al.^11^ reported that MoS_2_ FETs endured doses up to 37.5 kGy­(Si) without measurable performance loss. This resilience extends to other TMDs; Mondal et al.^8^ found that a PtSe_2_ humidity sensor exhibited identical sensing performance after a 10 kGy dose, and Ozden et al.^28^ reported only minor degradation in MoSe_2_ flakes at 100 kGy. Vogl et al.^29^ further confirmed that WS_2_ and MoSe_2_ FETs retain essentially unchanged performance after exposure to doses representative of Earth-orbit environments.

When degradation in TMD transistors is observed, the failure mechanism is consistently traced not to the TMD channel but to the accumulation of radiation-induced charges in the gate dielectric. Park et al.^23^ demonstrated that performance degradation in MoS_2_ phototransistors was primarily due to positive charge build-up in the SiO_2_ gate dielectric rather than catastrophic channel failure. Kim et al.^22^ confirmed this, finding that while the MoS_2_ channel mobility remained essentially unchanged after 3 kGy­(Si), the device showed significant positive threshold-voltage (V_th_) shifts, which were attributed to hole trapping within the Al_2_O_3_ dielectric. This V_th_ drift, caused by charge trapping at the TMD/dielectric interface, is the most commonly reported degradation pathway.

## Discussion

The preceding Results section synthesized the findings from 32 studies, organizing them by key thematic effects, such as defect creation, dose-dependence, spectroscopic signatures, emergent properties, and device performance. This thematic presentation revealed several clear trends but also highlighted apparent contradictions and nonlinear behaviors within the literature. This Discussion section now aims to critically analyze and interpret these findings. We will focus on resolving the mechanistic contradictions observed, evaluating the complex interplay of dose and environment, and identifying the critical gaps in the current research to propose a clear framework for future investigations in this field.

### Resolving Mechanistic Contradictions

The review highlights several apparent contradictions in the literature. Here, we propose explanations for these discrepancies that are crucial for building a unified model of radiation effects.

#### Doping Dichotomy: p-Type vs n-Type

Across the surveyed studies, the sign of γ-induced doping is jointly determined by the defect type and environment. In WSe_2_, both Ar and air exposures frequently yield n-type trends consistent with Se-vacancy donor states: PL spectra skew toward X^–^ trions and, where available, independent probes (work-function/KPFM, or XPS core-level/valence-edge shifts) corroborate electron accumulation.^36–40^ In contrast, S-based TMDs (MoS_2_, WS_2_) in air often display p-type signatures linked to oxygen involvement at S-vacancies: formation of Mo–O/W–O and oxide/interface states withdraws electrons and shifts the Fermi level toward the valence band.^21,33^ We emphasize that vacancy-driven n-doping dominates in inert ambients (vacuum/Ar) and when postexposure delay is short, whereas oxygenated/humid conditions and longer delays promote passivation/oxidation that can flip the polarity toward p-type. Accordingly, conclusions about doping require codiagnostics (e.g., KPFM/XPS or V_th_) in addition to PL.

#### Raman Puzzle: Conflicting Peak Shifts

Divergent Raman trends reflect a vector sum of three contributions: (i) doping, which primarily shifts A_1g_ (p-doping → A_1g_ blue-shift/stiffening; n-doping → A_1g_ red-shift/softening),^33,37^ (ii) biaxial/tensile strain, which predominantly red-shifts E_2g_^1^ (and to a lesser extent A_1g_),^24,32,41^ and (iii) disorder, which softens and broadens both modes.^32,34^ To clarify, we developed a ΔA_1g_–ΔE_2g_^1^ diagnostic map in Figure 1 and overlaid representative Raman shift data extracted from the literature, normalized relative to pristine (preirradiation) values. As shown in the map, the distance of the data points from the origin increases systematically with the severity of irradiation-induced modification, providing an intuitive measure of the overall impact of the total ionizing dose. Low-dose regimes cluster close to the center of the map, indicating weak perturbations of the lattice, whereas higher doses progressively drive the data toward the outer regions, reflecting stronger defect accumulation, chemical modification, and structural distortion.

**Figure 1:**
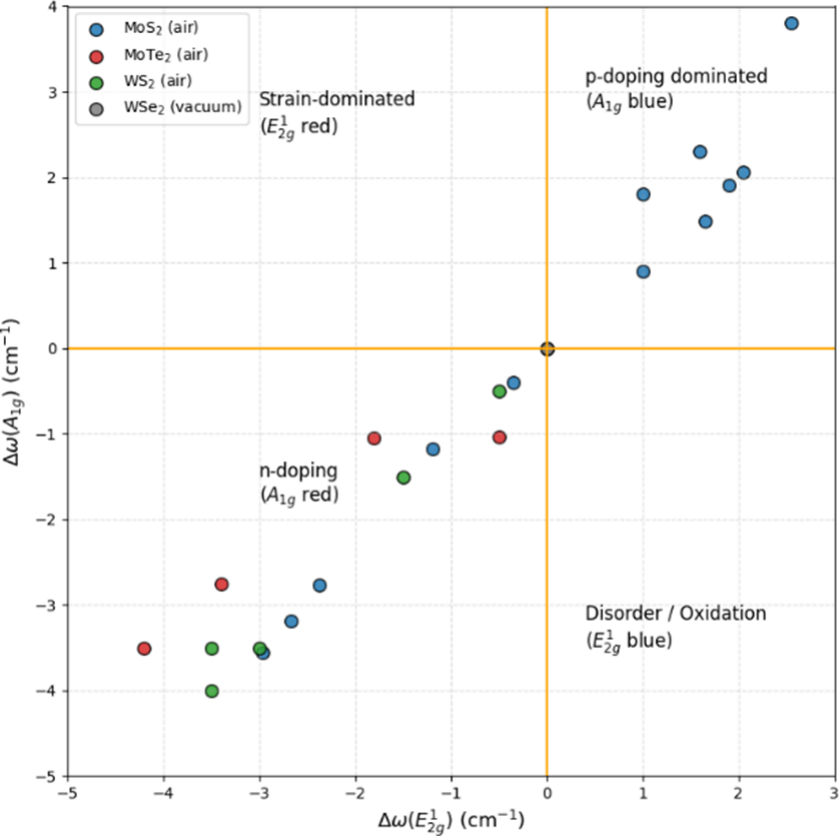
Raman diagnostic map correlating the irradiation-induced shifts of the in-plane E_2g_^1^ and out-of-plane *A*_1*g*_ phonon modes in transition-metal dichalcogenides (TMDs) under γ-ray exposure. Each data point corresponds to a literature-reported irradiation condition, for which both Raman modes were experimentally resolved and quantified. Data were compiled from studies on monolayer or few-layer MoS_2_,^13,25,27^ WS_2_,^34^ WSe_2_,^35^ and MoTe_2_.^41^ Studies reporting shifts in only a single Raman mode were excluded to ensure consistency and enable a direct comparison across materials and irradiation conditions.

Within this framework, the angular position in the map identifies the dominant physical mechanism. Cases exhibiting a positive ΔA_1g_ (A_1g_blue shift) with comparatively modest ΔE_2g_^1^ shifts are predominantly doping-driven, consistent with charge transfer effects that stiffen the out-of-plane mode. In contrast, reports showing concurrent red shifts of both A_1g_ and E_2g_^1^ modes, often accompanied by line width (FWHM) broadening, fall within strain- and disorder-dominated regimes, reflecting lattice distortion and defect accumulation.

Importantly, the map also reveals a dispersion of data points and several apparent outliers, which can be traced to differences in irradiation environment, dose history, and postirradiation exposure. These deviations do not undermine the diagnostic framework; rather, they emphasize its role as a first-order classification tool that captures the competition among doping, strain, disorder, and oxidation. Dose-dependent crossovers observed in multiple studies are naturally accommodated within this picture, where an initial doping-dominated response at low doses (e.g., A_1g_ blue shift) can progressively evolve toward strain- and disorder-dominated behavior at higher doses as vacancy density and chemical modification increase. In practice, a joint analysis of ΔA_1g_versus ΔE_2g_^1^, together with FWHM evolution and intensity ratios (I_A_1g__/I_E_2g_^1^_), and laser-power-dependent checks to exclude local heating, provides a robust pathway for disentangling irradiation-induced mechanisms.

### Reconciling Dose-Dependent Effects

The reviewed literature demonstrates that the material response to γ-radiation is highly nonlinear, where the TID can be a poor predictor of the outcome without considering competing, dose-dependent mechanisms. The effects are not always cumulative; in many cases, they exhibit “turn-around” behavior or clear activation thresholds.

A prominent example of nonmonotonic behavior is in defect-tuned functionalities. Dong et al.^20^ found an optimal dose of 100 kGy for enhancing the HER catalytic activity in MoS_2_, after which excessive disorder and oxidation caused performance to degrade. This “turnaround” effect is also seen in the electrical and optical properties. He et al.^27^ reported that the conductivity of MoS_2_ nanomaterials at room temperature peaked at a 10 kGy dose, decreasing at higher doses. Similarly, Garcia^16^ observed the MoS_2_ trion signal was maximized at 5 kGy, only to disappear at 40 kGy. Spectroscopically, Kolhe et al.^13^ captured this reversal, noting a Raman blue shift in MoS_2_ up to 300 kGy that inverted to a red shift at higher doses (500–800 kGy), indicating a transition from a doping-dominated regime to one dominated by strain and disorder.

In other cases, effects only manifest after a clear damage threshold is surpassed. Singh & Singh^25^ identified a “hardness window” for monolayer MoS_2_, finding it remained structurally intact up to ≈130 kGy, with progressive oxidation and etching appearing only beyond this point. A similar threshold for the onset of physical etching, driven by thermal strain, was observed around 50–100 kGy for WS_2_ and MoTe_2_.^32,41^ Saturation effects are also evident, as noted by Chen et al.,^11^ where V_th_ shifts in MoS_2_ FETs were pronounced during the initial 6.2 kGy­(Si) exposure but then saturated at higher doses.

This demonstrates a clear transition in the radiation–matter interaction: at low-to-moderate doses, irradiation acts as a tuning mechanism, while at high-TID regimes, it becomes a purely destructive mechanism. Doses from 400 to 1500 kGy consistently result in severe etching, sulfur sublimation, and the complete chemical conversion of the TMD into a metal oxide.

While TID is a primary variable, the effect of dose rate remains a critical gap in the literature. As shown in Table 1, the dose rates employed in the 32 reviewed studies vary by over 2 orders of magnitude (from ≈ 0.1 kGy/h to >20 kGy/h). Mechanistically, the rate should compete with oxygen diffusion and radiolysis kinetics: high-rate exposures may transiently favor bare-vacancy signatures (with postexposure oxidation catching up), whereas lower-rate exposures enable in situ passivation/oxidation during irradiation. However, no study performed a systematic comparison of a high-rate versus a low-rate exposure for the same TID. It is therefore unknown how competing processes, such as defect creation, in situ thermal annealing, and ambient chemical passivation, are influenced by the rate of energy deposition.

### Implications for Radiation-Hardened Electronics

The collective findings from the reviewed studies have significant and optimistic implications for the development of radiation-hardened electronics. The most critical insight is that in a well-fabricated device, the TMD channel itself is not the primary failure mechanism.

Several studies on MoS_2_ transistors consistently trace device degradation, such as threshold voltage (V_th_) shifts and mobility drops, to the accumulation of radiation-induced positive charges and hole trapping within the gate dielectric, specifically SiO_2_ or Al_2_O_3_. Park et al.^23^ and Kim et al.^22^ both demonstrated that even when device performance is compromised, the MoS_2_ channel remains largely intact. This suggests that the inherent radiation hardness of the 2D material often exceeds that of conventional 3D materials used to build the rest of the device.

This conclusion shifts the engineering focus from the 2D material to the device architecture and the dielectric interface. It presents a clear pathway for “radiation-by-design,″ where resilience is achieved by intelligently selecting and engineering the substrate and encapsulation layers.

Successful examples are already present in the literature. Park et al.^23^ showed that encapsulating a MoS_2_ phototransistor with a high-Z quantum-dot layer effectively mitigated dose effects by scattering incident photons, and Chen et al.^11^ demonstrated that using a polymer solid electrolyte (PSE) gate, which allows for trap saturation via preirradiation and thermal annealing, resulted in “large radiation tolerance” far exceeding typical SiO_2_-based devices.

Finally, this review indicates that irradiation should not be viewed only as a hazard but also as a potential postgrowth fabrication tool. The controlled, dose-dependent creation of defects has been shown to tune material properties in beneficial ways, such as inducing magnetism, optimizing catalytic activity, enhancing conductivity, and enabling ultrasensitive photon detectors. This opens a pathway for using targeted irradiation to selectively engineer device components or tune material properties with high spatial and dose precision.

#### Device-Level Degradation Pathways

Although several of the studies discussed below do not explicitly employ γ-ray irradiation, they interrogate the same ionization-driven physical mechanisms and therefore provide critical insight into the dominant device degradation pathways under ionizing radiation exposure.

Ionizing radiation can perturb TMD transistor operation through three concurrent pathways that should be distinguished to interpret “device tolerance” and to guide radiation-hard design. Importantly, a device may exhibit large electrical drifts even when the atomically thin channel remains largely intact, because the dominant sensitive volume for total-ionizing-dose (TID) response is often the surrounding dielectric stack rather than the 2D semiconductor itself.^42–44^

i Channel defect creation (intrinsic 2D damage). In the channel, energetic secondary electrons (for γ/X-ray) or direct particle collisions (for ions) can generate point defects (e.g., chalcogen vacancies, Frenkel pairs) that act as scattering centers and/or dopants.^44–46^ In transistors, this pathway primarily manifests as mobility/transconductance degradation, increased off-state leakage via defect-assisted transport, and changes in contact injection when defect creation extends into the contact region.^43,46^ Crucially, intrinsic channel damage can be isolated experimentally by decoupling irradiation of the flake from irradiation of the substrate/dielectric, enabling a more faithful estimate of “intrinsic radiation hardness” of the 2D layer.^44^
ii Interface-trap formation (TMD/dielectric interface and border traps). Ionization in the oxide and near-interface region can generate electrically active interface states and “border traps” that exchange charge with the channel on experimental time scales.^42–44^ This pathway typically produces strong degradation of subthreshold swing (SS), increased hysteresis, time-dependent recovery/annealing effects, and an apparent threshold-voltage drift whose sign can depend on trap occupancy and measurement conditions.^43–45^ A key practical point is that interface-trap contributions can dominate SS changes even when oxide charging alone would predict a different sign for ΔV_th_; near-interfacial oxide charges (border traps) can behave similarly to interface traps and therefore exacerbate SS.^42–45^

iii Bulk dielectric charging (oxide trapped charge). TID generates electron–hole pairs inside the gate dielectric; under typical bias fields, electrons are preferentially swept out while holes become trapped in the oxide bulk and/or drift toward the interface. The resulting fixed oxide charge alters the effective gate field and can dominate threshold-voltage shifts and leakage trends, with a strong dependence on dielectric material and thickness.^42–44^ Physics-based TID treatments for TMD FETs explicitly separate oxide-trapped charge density (often denoted N_ot_) from interface-trap density (N_it_), showing that (a) both scale with total dose, (b) thicker oxides tend to accumulate larger trapped charge densities, and (c) N_it_ strongly controls SS via an additional capacitance term, whereas N_ot_ can dominate surface-potential and ΔV_th_ shifts.^43^

Design implications and reporting guidance: Because the observed transfer-curve evolution can reflect a superposition of (i)–(iii), “radiation tolerance” should be reported together with the dielectric stack (material and thickness), biasing during irradiation, dose metric (e.g., krad­(Si) or kGy­(Si)), and postirradiation measurement timing (to capture relaxation/annealing).^44^ From a hardening standpoint, minimizing susceptible oxide volume (thinner oxides), selecting dielectrics with reduced hole trapping, improving interface quality/passivation, and reducing or eliminating oxide dielectrics in the gate stack (e.g., vacuum-gate paradigms) are all direct levers to suppress pathways (ii)–(iii) even when the channel is intrinsically robust.^42–44^

### Identified Gaps and Future Research Directions

This comprehensive review not only synthesizes the current state of knowledge but also illuminates critical gaps and apparent contradictions that must be addressed. The analysis of the 32 selected studies points to several key areas for future investigation.

- Decoupling Competing Mechanisms: This review identified clear contradictions, particularly in electronic doping (p-type vs n-type) and Raman shifts (red vs blue). These discrepancies are attributed to a competition between intrinsic defect-driven effects (e.g., vacancies) and extrinsic environmental effects (e.g., oxidation, strain). A significant gap exists in studies designed to decouple these mechanisms. Future research should involve systematic, parallel experiments that irradiate the same material on different substrates (to control strain) and in different environments (e.g., high vacuum, inert Ar, ambient air, and controlled humidity). This would definitely resolve whether the observed p-doping is an intrinsic or extrinsic oxidation effect and allow for a unified model of the competing influences on spectroscopic signatures.
- Investigating Dose Rate and Non-Equilibrium Effects: The literature focuses almost exclusively on TID, while the dose rate is completely unexplored. This is a critical gap, as the kinetics of defect creation, self-annealing, and chemical passivation are likely rate-dependent. While total ionizing dose (TID) is the dominant parameter reported in the literature, the dose rate is expected to play a decisive role by governing a competition between concurrent kinetic processes. At the microscopic level, γ irradiation continuously generates point defects (primarily chalcogen vacancies) through secondary-electron interactions, while, in parallel, several relaxation mechanisms may occur on comparable time scales: (i) chemical passivation of vacancy sites by oxygen or water in ambient environments, (ii) thermally assisted defect migration and recombination, and (iii) charge relaxation and detrapping in adjacent dielectrics. The balance between these processes is inherently rate-dependent.

At high dose rates, defect generation may transiently outpace passivation and annealing, favoring signatures associated with bare vacancies (e.g., intrinsic vacancy doping, sharper Raman shifts linked to lattice stiffening, or enhanced trion populations). Conversely, at lower dose rates, longer exposure times enable in situ oxidation, radiolysis-assisted chemistry, and partial self-annealing to proceed concurrently with defect creation, potentially leading to qualitatively different outcomes for the same final TID, including doping polarity reversal, suppressed defect-related PL features, or enhanced etching. This kinetic competition provides a natural explanation for the nonmonotonic and sometimes contradictory trends reported across studies that nominally reach similar doses.

As illustrated schematically in Figure 2, decoupling these competing pathways requires experimental protocols that go beyond the total ionizing dose alone. Future studies should therefore explicitly control and report: (a) the dose rate at fixed TID, to probe the balance between defect generation and compensating processes; (b) the irradiation environment (high vacuum, inert gas, ambient air, and controlled humidity), which governs in situ chemical passivation and radiolysis-assisted reactions; (c) biasing conditions during irradiation for device-based measurements, which critically affect charge trapping and transport in adjacent dielectrics; and (d) time-resolved postirradiation measurements to capture relaxation, detrapping, and delayed oxidation effects. Together, these controls enable a transition from purely dose-based descriptions toward a kinetic framework, allowing predictive modeling of radiation response in TMDs under realistic operational conditions.

- Expanding Material Scope and Systematic Comparisons: The research is heavily concentrated on MoS_2_ and WS_2_. While recent studies on WSe_2_, PtSe_2_, and MoTe_2_ have provided new insights, a systematic comparison is lacking. It is currently unknown how properties like chalcogen mass (S vs Se vs Te) or metal (Mo vs W vs Pt) fundamentally alter the response to irradiation. Future work should correlate these different TMDs side-by-side to build a comparative database.
- Advanced Defect Characterization and In situ Monitoring: Many of the conclusions about defect mechanisms are inferred from spectroscopy and supported by DFT. For example, the complex vacancies (V_1Mo+2S_ and V_1W+2S_) proposed to explain ferromagnetism have not been directly observed. There is a need for advanced postirradiation characterization, such as aberration-corrected scanning transmission electron microscopy (STEM), to directly image these atomic-scale defect complexes. Furthermore, nearly all studies are ex situ, meaning transient effects and immediate relaxation are missed. Future studies employing in situ monitoring (e.g., PL/Raman measurements during irradiation) are necessary to capture the real-time dynamics of defect formation and passivation.

**Figure 2:**
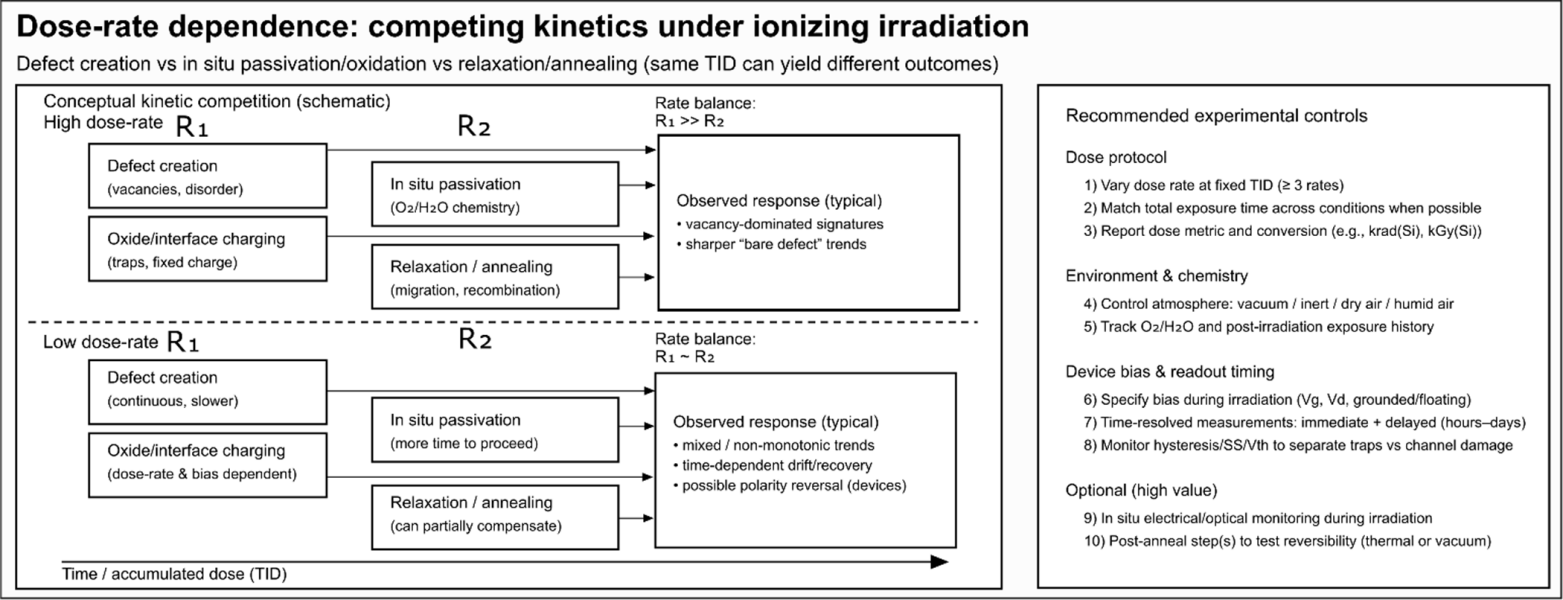
Conceptual roadmap illustrating dose-rate-dependent competing kinetics under ionizing irradiation in TMDs.

## Conclusions

This comprehensive review of 32 post-2016 experimental studies provides a unified framework for understanding the complex effects of γ irradiation on TMDs. Our analysis establishes that the primary defect mechanism is the creation of CVs. The subsequent material response, however, is critically dictated by the irradiation environment.

This environmental dependence resolves key contradictions in the literature: Intrinsic CVs, particularly in WSe_2_, act as n-type dopants, and extrinsic oxidation in ambient air, dominant in WS_2_ and MoS_2_, drives p-type doping via a charge-transfer mechanism.

This interplay among doping, strain, and disorder is reflected in the complex and often conflicting Raman shifts reported in the literature.

Furthermore, the material response to dose is highly nonlinear. This review identified clear “turn-around” effects, where properties like catalytic activity^20^ and electrical conductivity^27^ are optimized at moderate doses but are degraded by excessive disorder at higher doses. At extreme TIDs (e.g., > 400 kGy), irradiation becomes a purely destructive mechanism, leading to etching and complete oxidation.

For practical applications, this perspective yields two critical insights. First, γ-rays can be a precise defect-engineering tool to induce novel emergent properties, most notably RTFM, which is attributed to complex V_1M+2S_ vacancies.^17^ Second, the 2D TMD channel itself possesses an extraordinary radiation hardness. The primary failure mechanism in TMD-based electronics is consistently traced to charge trapping in the adjacent gate dielectrics (e.g., SiO_2_ or Al_2_O_3_).^22,23^

This shifts the engineering focus from the channel to the dielectric interface, opening a clear pathway for radiation-by-design. The identified gaps in the literature, particularly the unstudied effects of dose rate, the need for in situ monitoring, and the limited material scope, provide a clear roadmap for future research in this promising field.

## Methods

A systematic literature search was conducted to identify peer-reviewed studies examining γ-ray interactions with transition metal dichalcogenides. Four electronic databases—Web of Science, Scopus, IEEE Xplore, and Google Scholar—were searched using combinations of the terms “γ radiation”, “gamma irradiation”, “gamma”, “gamma-ray”, “γ radiation”, “γ irradiation”, “γ”, “γ -ray”, “transition metal dichalcogenide”, “TMD”, “MoS_2_”, “MoSe_2_”, “WS_2_”, and “WSe_2_.” Searches were limited to articles published in English from 2016 to 2025.

After all records were exported into a reference manager, duplicates were removed. Titles and abstracts were screened for relevance to the γ-ray effects on TMDs. Then, the full texts of potentially eligible articles were assessed according to predefined inclusion criteria.

Data extraction from the final set of studies included material, form factor (exfoliated flakes, films, devices), radiation source, total dose, irradiation environment, and key findings on defect formation, carrier dynamics, and device performance.

For consistency and cross-study comparison, all reported radiation quantities were standardized to the total ionizing dose in kGy, dose rate in kGy h^–1^, and source activity in GBq throughout this work.

## Abbreviations

2D: two-dimensional
2DM: two-dimensional material
Ar: argon
CV: chalcogen vacancy
DFT: density functional theory
FET: field-effect transistor
FTO: fluorine-doped tin oxide
fwhm: full width at half-maximum
HER: hydrogen evolution reaction
KPFM: Kelvin probe force microscopy
MCNP: Monte Carlo N-Particle (code)
MoS_2_: molybdenum disulfide
MoSe_2_: molybdenum diselenide
MoTe_2_: molybcadenum ditelluride
PL: photoluminescence
PSE: polymer solid electrolyte
RH: relative humidity
RTFM: room-temperature ferromagnetism
SiO_2_: silicon dioxide
STEM: scanning transmission electron microscopy
TID: total ionizing dose
TMD: transition metal dichalcogenide
WS_2_: tungsten disulfide
WSe_2_: tungsten diselenide
XPS: X-ray photoelectron spectroscopy
γ: gamma
